# An mHealth App-Based Self-management Intervention for Family Members of Pediatric Transplant Recipients (myFAMI): Framework Design and Development Study

**DOI:** 10.2196/32785

**Published:** 2022-01-04

**Authors:** Riddhiman Adib, Dipranjan Das, Sheikh Iqbal Ahamed, Stacee Marie Lerret

**Affiliations:** 1 Department of Computer Science Marquette University Milwaukee, WI United States; 2 Department of Pediatrics Medical College of Wisconsin Milwaukee, WI United States

**Keywords:** pediatric patients, transplant, mobile health, mHealth, family self-management, smartphone

## Abstract

**Background:**

Solid-organ transplantation is the treatment of choice for children with end-stage organ failure. Ongoing recovery and medical management at home after transplant are important for recovery and transition to daily life. Smartphones are widely used and hold the potential for aiding in the establishment of mobile health (mHealth) protocols. Health care providers, nurses, and computer scientists collaboratively designed and developed mHealth family self-management intervention (myFAMI), a smartphone-based intervention app to promote a family self-management intervention for pediatric transplant patients’ families.

**Objective:**

This paper presents outcomes of the design stages and development actions of the myFAMI app framework, along with key challenges, limitations, and strengths.

**Methods:**

The myFAMI app framework is built upon a theory-based intervention for pediatric transplant patients, with aid from the action research (AR) methodology. Based on initially defined design motivation, the team of researchers collaboratively explored 4 research stages (research discussions, feedback and motivations, alpha testing, and deployment and release improvements) and developed features required for successful inauguration of the app in the real-world setting.

**Results:**

Deriving from app users and their functionalities, the myFAMI app framework is built with 2 primary components: the web app (for nurses’ and superadmin usage) and the smartphone app (for participant/family member usage). The web app stores survey responses and triggers alerts to nurses, when required, based on the family members’ response. The smartphone app presents the notifications sent from the server to the participants and captures survey responses. Both the web app and the smartphone app were built upon industry-standard software development frameworks and demonstrate great performance when deployed and used by study participants.

**Conclusions:**

The paper summarizes a successful and efficient mHealth app-building process using a theory-based intervention in nursing and the AR methodology in computer science. Focusing on factors to improve efficiency enabled easy navigation of the app and collection of data. This work lays the foundation for researchers to carefully integrate necessary information (from the literature or experienced clinicians) to provide a robust and efficient solution and evaluate the acceptability, utility, and usability for similar studies in the future.

**International Registered Report Identifier (IRRID):**

RR2-10.1002/nur.22010

## Introduction

Solid-organ transplantation is the treatment of choice for children with end-stage organ failure [1,2], such as heart, lung, liver, kidney, pancreas, and small bowel failure. Although transplantation is now a routine surgical procedure throughout hospitals in the United States, it is only a treatment, not a cure. In 2020 alone, approximately 2000 children in the United States received a transplant [3]. The benefits of transplantation are limited by risks of the immediate procedure and chronic immunosuppression [4,5], making the posttransplant period critically important. Posttransplant challenges include the provision of focused discharge plans, family functioning, and family member roles after transplant, which are continuously being explored for pediatric transplant recipients [6-8].

The at-home daily management of posttransplant pediatric patients is of utmost importance and can impact patient and family coping [9]. Families must learn to adjust to multifaceted lifestyle changes and care, such as precise administration of multiple medications, management of abdominal drains and enteral tube feeding, or central line care. These actions, along with planning of follow-up care for laboratory studies and clinical appointments, make family life complex. These challenges are some of the factors that may place patients at risk for readmission in the first 30 days after hospital discharge [10,11]. Parents of pediatric liver and kidney recipients with higher stress levels (self-reported) find greater difficulty with medication administration [12,13]. However, frequent and focused communication and support from the health care team improves postdischarge health outcomes in different patient groups, including patients with heart failure [14], acute kidney injury [15], and type 2 diabetes [16]. As self-management strategies using a mobile health (mHealth) intervention improve overall health outcomes for adult lung transplant patients [17], the same should hold true for pediatric transplant patients.

Considering current technological advancements globally, researchers have been exploring the potential of commonly available smartphone apps for the provision of better and improved health care and support [18-23]. For medically complex adult patients, regular contact and communication through the use of smartphone apps improve general health conditions [24,25]. This creates a provision for providing mHealth interventions through effectively designed smartphone apps that support the interactive partnership between family members and the health care team [26]. A few examples include improvement of well-being [27,28], obesity treatment [29,30], and recovery from alcoholism [31,32]. In addition, patients undergoing transplantation have been identified as an ideal population to utilize and perform mHealth research [33]. Therefore, this is an opportunity to build a smartphone-driven app framework to provide connected health management solutions and support family members of pediatric transplant recipients.

This paper reports the outcomes of the design and development of a smartphone-based intervention app for an mHealth family self-management intervention (myFAMI) for families of pediatric transplant recipients. The research project was initiated by a collaborative team of health care providers, pediatric nurses, and computer scientists. The myFAMI protocol is described in the protocol manuscript [34]. The features and functionalities of the developed myFAMI smartphone app and associated software framework are reviewed. We outline the key points in designing such an app, the challenges, and the strategized development procedure. The motivation for this project was to leverage the usage of consumer digital health care and its potential for supporting transplant families after hospital discharge. myFAMI was designed to support the exchange of information between the caregiver (family) and the health care team (nurses). The aim of this research is to use the action research (AR) methodology to build a well-designed and evidence-based mHealth intervention app (myFAMI).

## Methods

The myFAMI software framework was designed using the AR methodology [35,36] based on prior research motivations and completed through separate stages of research and development: research discussions, feedback and modifications, alpha testing, and deployment and release improvements.

### Design Motivation

The initial discussion for provision of the intervention for family members included building cellular text-based information provision. Although the usage of cellular text seems feasible and reliable, it was not comprehensive to complete the full list of our requirements for this research project (ie, capture of survey completion time, assure individual notification receipt). Lessons from previously completed text-based interventions [37,38] helped the research team plan for commonly known issues. The research team collaborated biweekly over 3 months to discuss all functionalities, use cases, user bases, and the timeline.

The collaborative research team comprised health care providers, nurses, and computer scientists. The team planned a randomized controlled trial (RCT) with myFAMI [34] as the treatment and standard postdischarge follow-up care as the control. The intervention promoted daily communication, facilitated through a smartphone app, which led the research team to the design and development of such an app framework. Family units of pediatric transplant recipients (heart, kidney, liver transplants) from four major US-centered pediatric transplant programs were recruited for the study. Inclusion and exclusion criteria were used to select a bias-free population group and were randomized to reduce bias [34].

The research problem for this part was to develop a software framework that was:

- Available for all (most) smartphone users (Android and iOS)
- Available for the survey submission (within the time frame)
- Able to notify users for survey submission through smartphones
- Technically error free (minimal to no bugs in the software)

The primary design motivations were:

- Easier connectivity with the nurses: Family members needed to connect with the nurses on a daily basis, and the primary point was to have easier and fluent connectivity with nurses.
- Interesting and colorful: To avoid monotony, the app needed to feel interesting and look colorful to the end users.
- Error free and easy to use: Since the intervention is technology assisted, getting an error during live use would add bias to the study results. For this reason, the app needed to be absolutely error free and intuitive to use.

Although the focus was on the end users, that is, family members of pediatric patients, the framework was developed in such a way that nurses and system admins could easily use and navigate the system with personalized access. The set of requirements defined the ideal app for this purpose and guided the research team in designing the final framework.

### Action Research Methodology

The AR methodology [35,36] was adopted for the design of the myFAMI framework to support the collaborative research between a team of providers, nurses, and computer scientists. The AR methodology [39,40] is the study of technology, its applications in the real world, and the practical real-world consequences of those technology-assisted actions. Our research aimed to follow the set of research approaches under AR, with a focus on technology design.

Within AR, the researcher tries to provide a service to a research client (in our case, nurses), as well as simultaneously add to the knowledge in that particular domain (in our case, discharge support in pediatric solid-organ transplantation patient family members). AR aims to both improve the client of the study as well as obtain further insights into the design, which is one of the key appeals of AR. As described in detail in the Results section, myFAMI was designed with 3 level of access roles to the app framework, and specific functionalities in each access were curated with care from individuals within that access level. Upon collection of requirements, the research team had recurring meetings to decide on a set of features that is feasible within the development timeline yet significantly boosts the productivity of the complete procedure with minimal steps. All the webpages and app pages went through multiple iterations of feedback, and following the AR methodology, changes were made based on that feedback.

Within the app design phase, the key idea was to focus on the ease of use of the technology. Any research team can prototype an idea and build an app framework; however, the framework will only become useful and meaningful when it is used in practice, and AR focuses on practice.

### Research Stages

**Figure 1:**
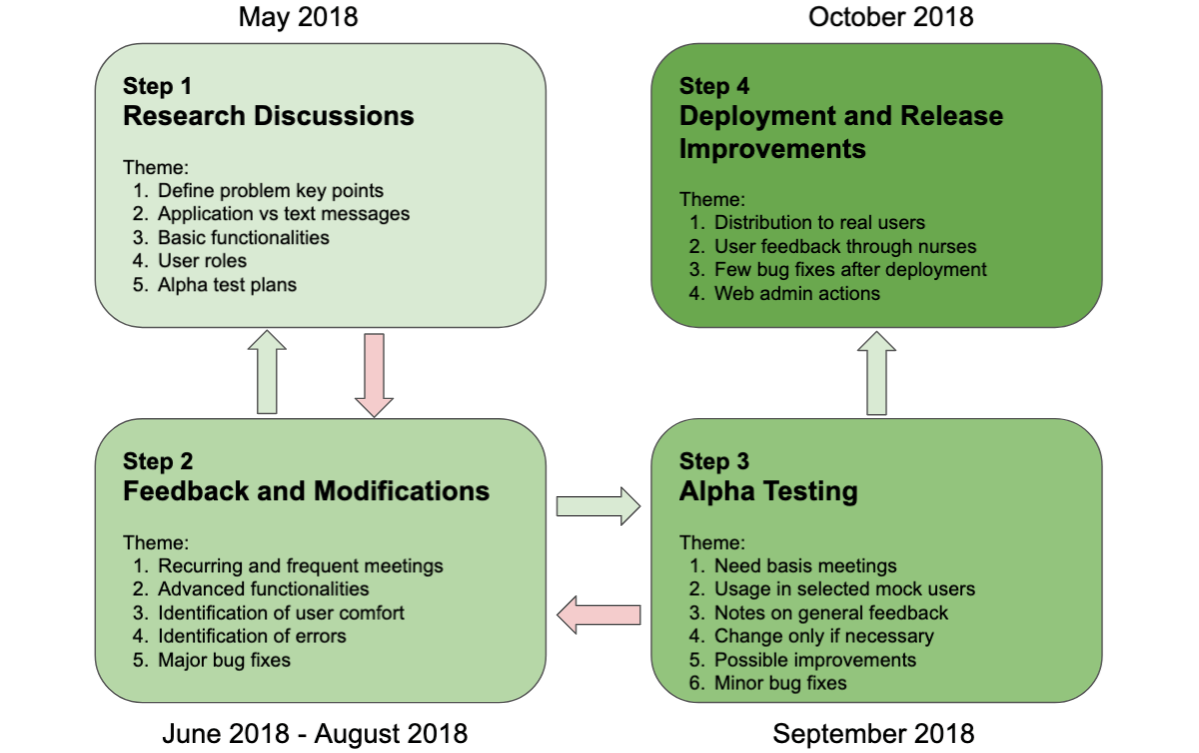
Workflow and timeline of the design and development process of the myFAMI app framework. Bidirectional arrows represent moving back and forth between stages, and unidirectional arrows represent moving forward with time. myFAMI: mHealth family self-management intervention.

A multidisciplinary research team, including health care providers, pediatric nurses, and computer scientists, worked collaboratively to conceptualize and generate the smartphone app framework. Figure 1 portrays the workflow of the development process, that is, the workflow and timeline of the design and development process of the myFAMI app framework.

#### Stage 1: Research Discussions

Based on the literature review, expertise-based knowledge of pediatric transplantation and previous experience of the development of app frameworks, the research team initiated relevant research discussions. The primary focus of these discussions included deciding on the most important features, defining the problem key points, laying out basic functionalities, deciding on the best intervention method (text message vs smartphone), confirming user roles, and laying out alpha testing plans and timelines. A limited number of research meetups were conducted to decide on the key points in this context.

#### Stage 2: Feedback and Modifications

In the next stage, the computer scientists in the team started building the minimum viable product (MVP) [41], while the nurses continued to evaluate it. At this stage, the team had more frequent meetings and decided on advanced functionalities for the app. Transplant family members were not involved in this stage; however, they contributed to the final shape of the MVP in the later stage of development (stage 4). The meeting discussion points frequently moved back and forth between stages 1 and 2 and explored potential factors to improve efficiency, user comfort features, and potential errors. This led to some major bug fixing on the app framework.

#### Stage 3: Alpha Testing

In this stage, the major task was to test the apps for any potential bugs or errors. The nurses took turns in role-playing and went through each section of the apps. A few issues were identified: (1) A check for enabling in-app notifications was needed in case any notification through the apps was missed; (2) test pagers were missed a few times, which was resolved promptly; and (3) the slider for survey response in the iOS app was accidentally set at 2 at startup, which was fixed. All the issues were resolved to prepare the framework for final deployment. The research team also decided on ways to download user data and portray them in the admin panel at this stage.

#### Stage 4: Deployment and Release Improvements

After completion of the alpha testing stage, the myFAMI app framework was deployed in the field. Although the initial deployment to the real world (running the public server, releasing the app in the respective app store, ie, Google Play Store and Apple App Store) was completed at this stage, access to the participants continued over time based on the timeline of the intervention protocol. Minor changes were made at this stage based on feedback from the 2 participating transplant families (as beta testers) of the trial: (1) In the earlier version, the word “emesis” was used and changed to “vomiting” in later releases, and (2) some other texts required rewording to properly express the true content, such as the notification response “You should expect a phone call” was changed to “Thank you for your participation. You may be contacted by the study team to discuss one or more of your concerns.” These minor changes significantly improved the overall user experience and concerns expressed by the participants.

### System Component Description

#### Users and Their Roles

The target audience of the intervention app was family members of pediatric transplant patients. From the app development perspective, to make the intervention effective, we included 3 types of users in the app ecosystem:

- Participants: By “participant,” we refer to the family members of the pediatric transplant patients. Since they were “participating” in this intervention, we used this term, contrary to other options, such as “patients” or “users.” Participants only had access to the smartphone apps and were unaware of the other components. The primary role of the participants was to check in daily in the myFAMI smartphone app and to complete the required survey on the pediatric transplant patient’s health status and coping difficulty.
- Nurses: Nurses in the hospitals were one of the key user groups in our framework. Nurses were primarily responsible for responding to trigger alerts by the participants. Because of this, nurses’ access was limited only to the myFAMI web admin panel.
- Superadmin: On the highest level, the superadmin had access to the database itself and app codes required for the registration of participants to the myFAMI smartphone app. The superadmin access was limited to a small number of people, the project principal investigator (PI), and the app management team. The research assistant and the PI’s role was to preregister the participants through the superadmin access. The superadmin access was provided through the admin panel generated by the Django web framework (Django Software Foundation), and it was minimally used except in cases of emergencies.

#### App Components and General Functionalities

myFAMI is a multicomponent, user-centered app with multiple modular fragments efficiently tied together and available on both Android and iOS platforms. The prime components of the framework are:

- The web app (for nurses’ and superadmin usage) with Application Programming Interface (API) endpoints (for web server connectivity with the smartphone app)
- The smartphone app (for participants’ usage).

The participants are registered through the web app and use the smartphone app, which stores and retrieves information on the web server, as required.

The process starts when a family agrees to be a participant in the study after discussion with the physician. The participant is registered by the research assistant or the PI through the myFAMI web admin panel. Each participant’s personal information is stored privately within REDCap [42], where only the research team has access to it. Individually, each participant is connected through a unique “study id,” which is only known to the nurses and unknown to the participants. While registering to the system, the participants are also provided access to the myFAMI smartphone app, although its access is not granted until the patient is released home with the family.

**Figure 2:**
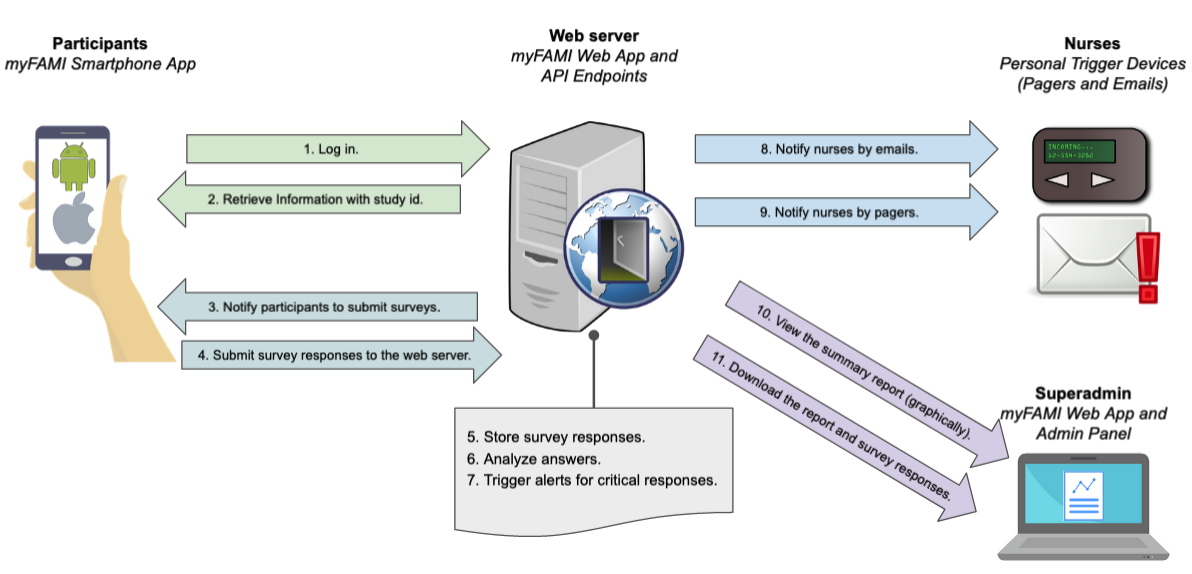
Interaction between app components and users' actions. API: Application Programming Interface; myFAMI: mHealth family self-management intervention.

A general overview of the day-to-day operation of the myFAMI app ecosystem is shown in Figure 2. It portrays step-by-step communication stages between the participants, the web server, nurses, and the superadmin.

Per protocol of the designed intervention (described in detail in Lerret et al [34]), at day 1 after hospital discharge, the myFAMI participants (intervention group) are granted access to the myFAMI smartphone app through the myFAMI web app. The participants log in to the myFAMI smartphone app, and their specialized access token is stored on the smartphone. This token is used throughout the study to authenticate their identity and access to the server. Every day at 8:00 AM local time, the web server pushes notifications to the participants for a reminder to submit survey responses. After the participants submit their responses, the response is sent to the web server, stored, and further analyzed to identify the immediate potential problem. If a potential problem (eg, serious deterioration of health outcome measures) is identified, the web server automatically triggers an alert notification to the nurse team. This alert contains information about the issue for the specific participant. The nurse then responds by calling the participant within 2 hours to discuss the potential problem. If no alert is triggered, no action is taken, and the response is simply stored in the web server for later reviewing by the research team.

Based on the action cycle and study protocol planned, a set of basic requirements were decided by the collaborative research team for the myFAMI app ecosystem. The goal was to build all the basic functionalities, distribute the functionalities as best fitted, and later improve based on discussions in iterations. The basic requirements discussed were:

- Participant actions:
  Register and log in.
  Receive a notification for a reminder.
  View, answer questions, and submit the survey.
- Research team actions:
  Log in to the admin panel.
  Register new participants.
  View participant details, survey submission dates, and individual survey responses.
  Download and visualize survey response data.
  Update alert trigger communication media: pagers, phone numbers, or emails.

#### Factors to Improve Efficiency

One key feature of our study was a focus on specific components that would add ease of access and usability for the participants using the myFAMI smartphone app. Any generic smartphone app would be able to collect relevant data needed; however, in many studies, we face the issue of missing data [43]. A main reason for this issue is the lack of human-centered design in smartphone apps [44] and apathy toward responding correctly. To mitigate these issues and to motivate the participants to regularly and easily submit responses, we focused on a few factors that improve overall efficiency. These features included improvements to the participant experience to make it easy and intuitive to take actions in the app. In our case, we improved on the following:

- Personal encouragement messages: To avoid monotonicity of daily push notifications in the myFAMI smartphone app, we added a variety of motivating text messages. Personalized text messages have been shown to improve the overall user response in smartphone interventions [45,46]. The personalized messages in myFAMI are related to the number of days passed past discharge and adding a personal tone to the scheduled notifications. A few examples are:
  “Welcome to Day 1 of the transplant study. Please complete the questions on the app by 10:00 AM today. Thanks for your participation.”
  “Good morning! Due to popular demand, we have your questions for today! Please view now.”
  “Only 1 more week to go! Check out today’s questions! Thanks for helping with this study.”
- Clean and clear progress bar: During early testing of the myFAMI smartphone app, we explored test users not answering all questions, because of not remembering how many questions need to be answered in a complete survey daily. In the first phase, we added a textbox saying “Question 1 of 8.” However, it still did not change much, since users do not always read all the texts. Since a visual cue is better than a textual cue [47], we added a clear progress bar along with that, which visually shows how much of the survey is left. This feature helped participants in getting an idea of the end line for the daily surveys.
- Easily interpretable responses: In specific questions (eg, “Does your child have a fever?”), when the participants responded “Yes,” we aimed to ask them additional questions on the actions taken. This is part of the train-up completed prior to releasing, and since this is a standard follow-up question, asking about it helps the nurses make a decision and reach back to the participants with further information. In its response, that goal was to incorporate easy-to-understand responses that make the survey submission more personal and like a conversation. A few examples are:
  “I have not done anything different.”
  “I have given a medication.”
  “I have changed the diet.”

## Results

### Principal Findings

Here, we describe the complete end product of the myFAMI app as a result of the AR methodology maintained through our design and development process. First, we discuss an overview of the app framework, followed by a description of 2 complementary app components, the admin web app with API endpoints and the smartphone app. Our final product, as targeted, successfully presents myFAMI and works great as a communication bridge between family caregivers (participants) and nurses.

### Web App and API Endpoints

The web app (for nurses’ and superadmin usage) with API endpoints (for web server connectivity with smartphone apps) is the main power source of myFAMI. The web app runs directly on the web server and connects the myFAMI smartphone app with the web server through API endpoints. API endpoints signify a set of connections between computer programs, more commonly a software interface to connect with another software [48]. The myFAMI web app is written in pure Python language on the Django web framework [49]. The Django web framework is wildly popular in the software development community and has been used to develop popular web services [50], such as Instagram, Mozilla, and Pinterest. A few benefits [49,50] of the Django web framework to build web apps are as follows: (1) Django provides an easy user authentication protocol, (2) Django has built-in admin panel support for easier and faster development, and (3) Django follows the model-view-template (MVT) pattern and handles the controller by itself, allowing developers to easily code and prototype with fewer errors.

The primary focus of the myFAMI web app is threefold: (1) provide access to daily operation for nurses, (2) provide access to superadmins for smooth operation and overview, and (3) keep API endpoints running for the myFAMI smartphone app. We broadly categorize and describe specific features for individual groups within the myFAMI web app.

**Figure 3:**
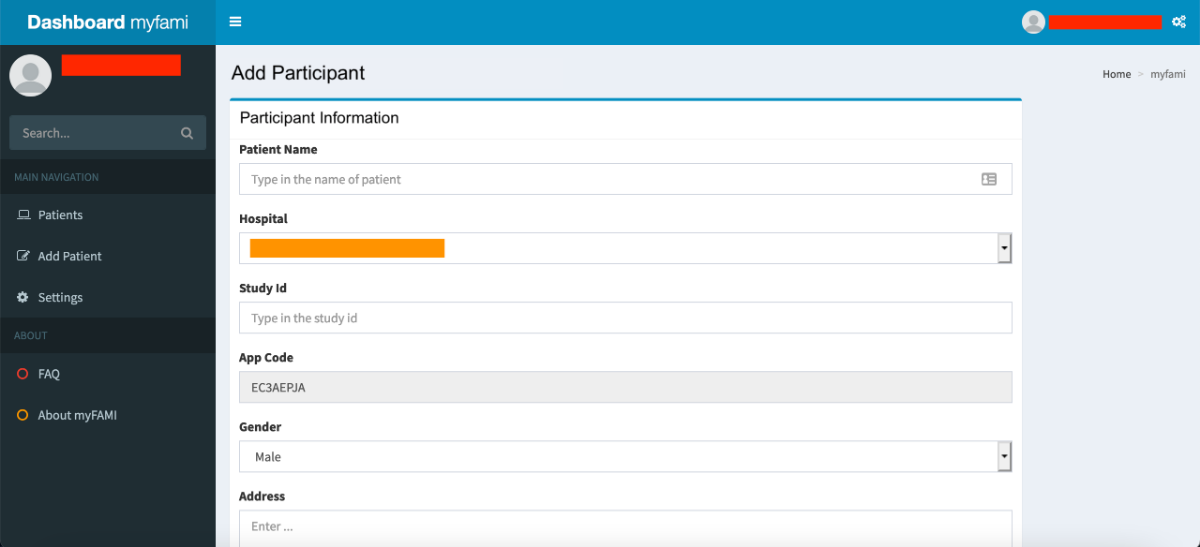
Web app screenshot: registration of new participants in the myFAMI web app. myFAMI: mHealth family self-management intervention.

**Figure 4:**
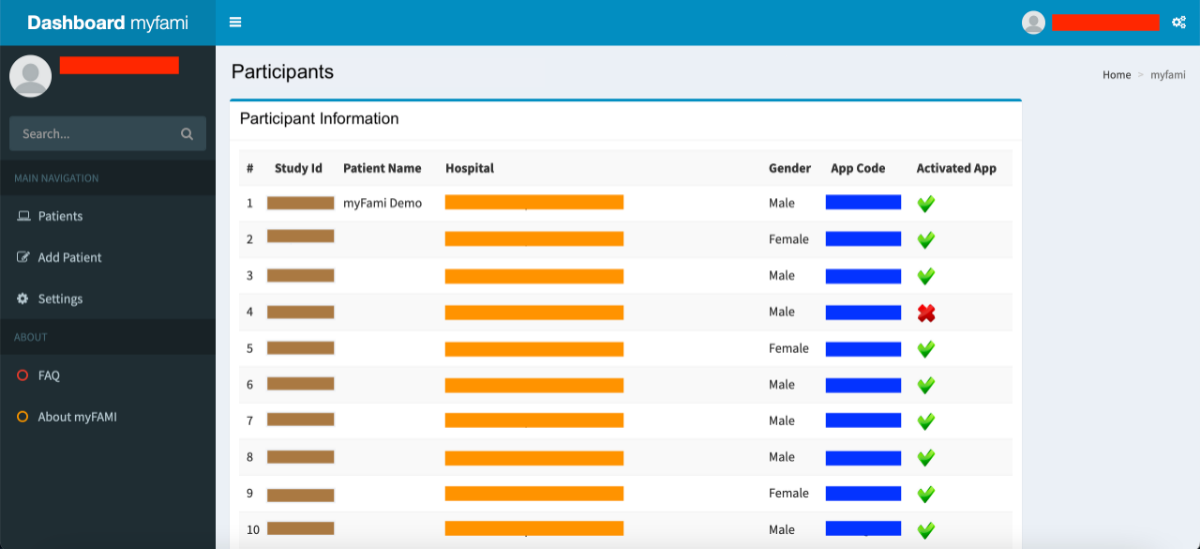
Web app screenshot: dashboard after log-in by researcher. myFAMI: mHealth family self-management intervention.

**Figure 5:**
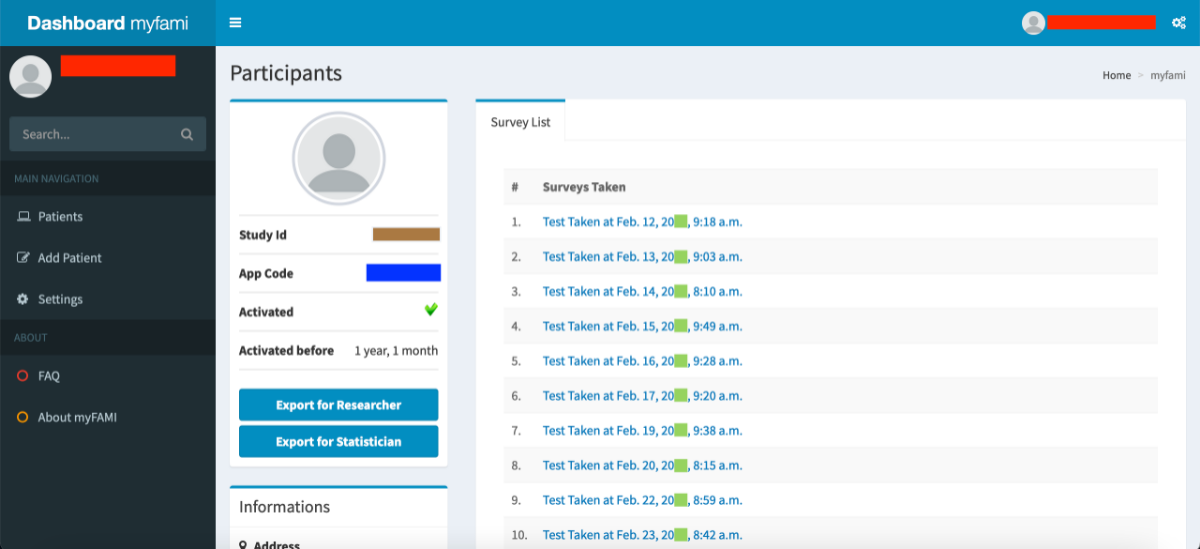
Web app screenshot: specific participant details. myFAMI: mHealth family self-management intervention.

**Figure 6:**
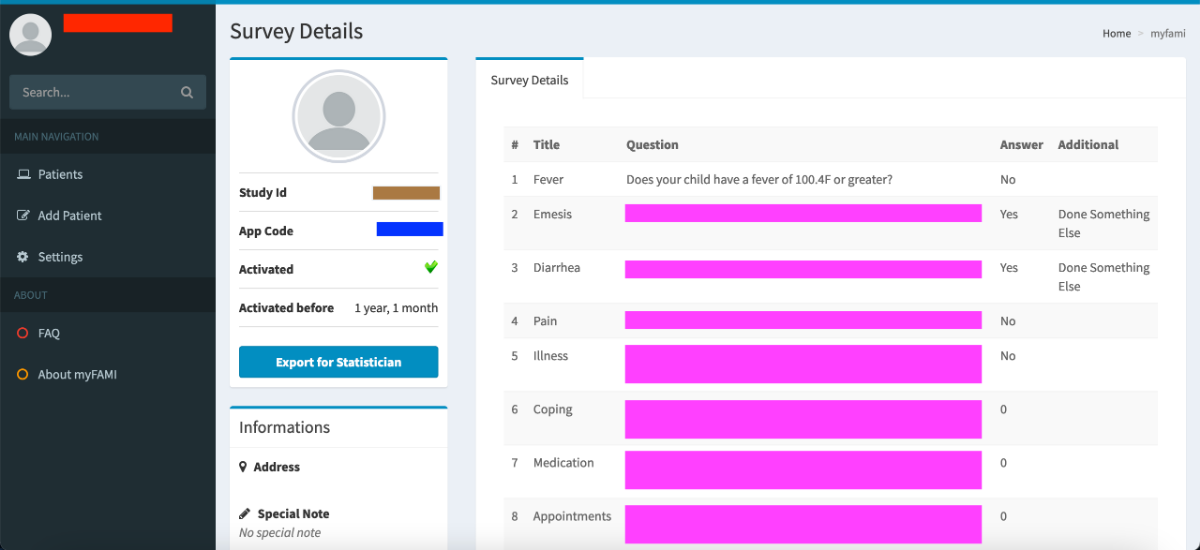
Web app screenshot: single survey response by a single participant. myFAMI: mHealth family self-management intervention.

**Figure 7:**
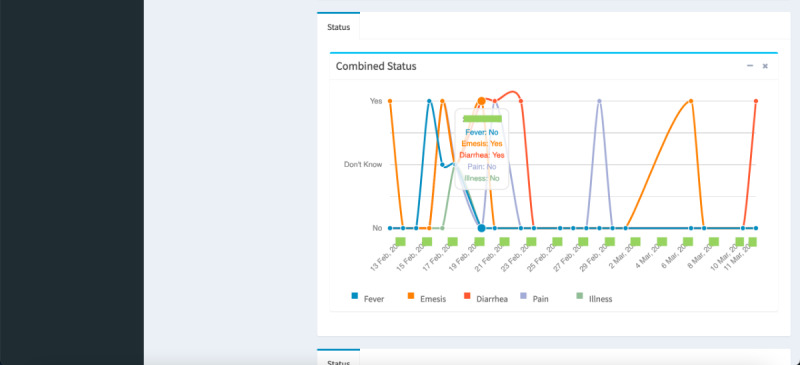
Web app screenshot: graphical summary of all survey responses by a single participant. myFAMI: mHealth family self-management intervention.

**Figure 8:**
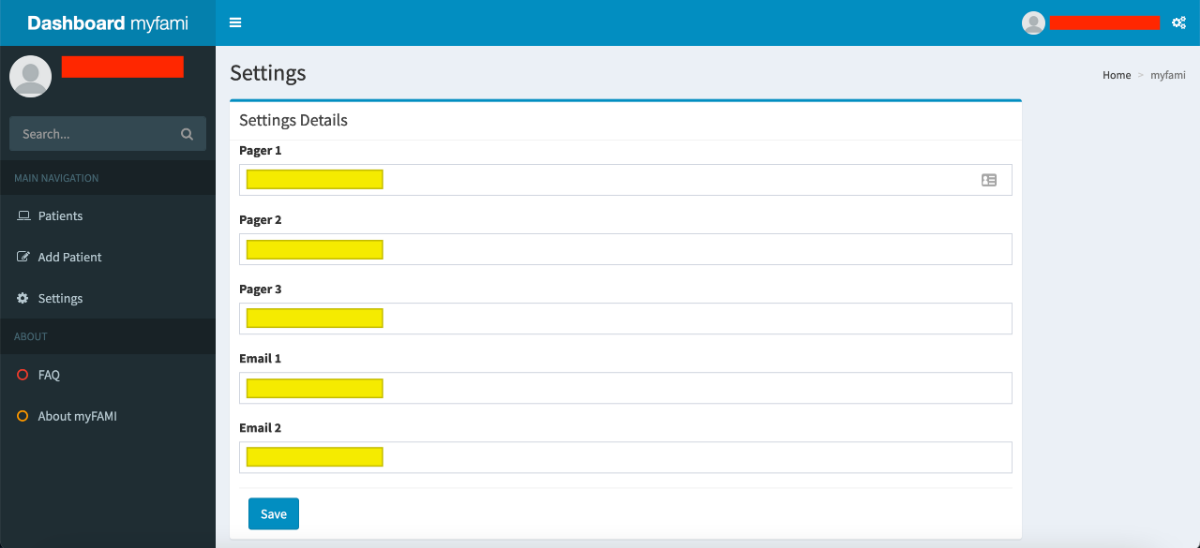
Web app screenshot: update page for communication endpoints (pager numbers and emails). myFAMI: mHealth family self-management intervention.

Screenshots of individual modules of the myFAMI web app are presented in Figures 3-8, along with a brief discussion below. Personalized information is redacted through color coding (red represents the myFAMI web app username, orange represents personalized information by participants, blue represents a unique appcode for participant access control, brown represents a unique study id for the participant, purple represents original survey responses, green represents true time, and yellow represents communication endpoints).

For the superadmin access level, the main features of the myFAMI web app are based on component creation:

- Registration of a new hospital center: We started participant registration at 1 major US pediatric transplant center and later added 3 other large centers for the study. The centers are located in different time zones, so the superadmins had to create “Hospital” entities through the myFAMI web app along the studies. The time zone was significant since it allowed us to notify participants from different locations to get smartphone notifications daily at the same time (8:00 AM).
- Registration of a new nurse: Several registered nurses were employed for the study. One of the key features of the myFAMI web app is to register new nurses to the system and provide them credentials so that they can log in to (and log out of) the myFAMI web app, as well as start receiving trigger alerts (discussed later in the Smartphone App: Android and iOS section).
- Update communication for alerts: The myFAMI web app also has a Settings page for communication endpoints. Two email and pager numbers for the PI and the team of research nurses are set for receiving the triggers, and the numbers are not hard-coded but are rather set up as a changeable field for flexibility and easier adjustments driven by the study (Figure 8). The trigger emails/texts contain specific information about the participant survey responses.

We broadly categorized the features in the myFAMI web app for nurses into 4 groups:

- Registration of participants: Nurses can log in to the myFAMI web app to register new participants (Figure 3). They can also enable/disable the app access from their end, as well as register participants to receive daily notifications for the smartphone app.
- View participants and survey responses: Nurses (and researchers) can obtain an overview of the total list of participants under individual study centers (Figure 4) and also go to a specific participant’s profile (Figure 5). The option to view individual survey responses (Figure 6) is also available to make better inferences about the next course of action.
- Overview of data: For researchers, we included 2 individual options to download the aggregated dataset from the admin panel. One download option was for researchers where the responses were stored as plain text, and the other download option was for the statistician where the datasets were coded by numbers (along with a codebook provided). This was helpful since data could be accessed and visualized while the study was ongoing.
- Graphical representation: For data visualization, a graphical overview of all survey responses by a single participant (Figure 7) is provided in the myFAMI web app. The feature is immensely helpful and helps nurses obtain a quick overview of the past status of the pediatric transplant patient at home.

Finally, we list selected significant API endpoints with actions on the web server as well as the smartphone, and their rationale as well, as shown in Table 1.

**Table 1:** Web API^a^ endpoints with functionality description, rationale, and action on smartphones.

API endpoint	Functionality	Action on smartphone apps	Rationale
<base_url>/appcode/verify/<str:appcode>^b^	Verify appcode	Activates a specific user based on the appcode	After installing the myFAMI^c^ smartphone app, participants are authorized for the survey through a unique appcode. The API endpoint validates individual appcodes and stops intruders/accidental users who get access to the myFAMI app through app stores.
<base_url>/token/upload/<str:platform>/<str:appcode>/<str:token>	FCM^d^ token upload with platform information (iOS/Android)	Collects a unique FCM token from the smartphone and uploads it to the web server	The token is used by the FCM service to send daily notifications to the appropriate recipient. The API endpoint uploads a unique FCM token to the myFAMI web server.
<base_url>/user/verify	User authentication	Collects log-in credentials and checks with the web server	The API endpoint checks log-in credentials of the participants and allows them access to the myFAMI smartphone app.
<base_url>/survey/save/<str:appcode>	Save survey responses	Saves the daily survey response	The API endpoint stores daily survey responses by participants and stores them on the web server.
<base_url>/send/notification	Send push notifications from the FCM server to the myFAMI smartphone app	When a push message is received from the FCM server, shows the notification to the participant using the app	When called, the API endpoint pushes a notification prompt to the FCM server, which pushes a notification to the individual’s smartphone. Participants in the intervention group received daily notifications for 30 days.

^a^API: Application Programming Interface.

^b^The “str” in the endpoint signifies a string code in the codebase.

^c^myFAMI: mHealth family self-management intervention.

^d^FCM: Firebase Cloud Messaging.

### Smartphone App: Android and iOS

Although the myFAMI web app keeps running in the background for smoother operation of the intervention, the myFAMI smartphone is the user interface that the participants respond to and interact with. The myFAMI smartphone is built for 2 individual platforms, Android and iOS, to cover the broad range of smartphones the participants might have. For easier availability, the apps are made available in both app stores (Google Play Store and Apple App Store). Android and iOS are the two most popular smartphone operating systems currently and hold over 99% of market users as of 2021 [51]. In case a participant did not have any of these 2 types of smartphones, they were provided with one with the myFAMI app installed.

Both versions of the myFAMI smartphone app are identical and built with native code support, in Android Studio using the Android software development kit (SDK) [52] with Native Java as the programming language [53] and in XCode [54] with Swift programming language [55]. Both the programming languages are wildly adopted in the programming world, and the SDKs have been maintained for a long time by the Android community and Apple.

The prime feature of the myFAMI smartphone app is easier connectivity with the web server, through (1) a reminder to complete the survey questions with daily notifications sent from the web server and (2) capturing of the user response to the survey and storing it on the web server.

For the smartphone apps, we summarize the prime features:

- Participant access: The participants can log in to the myFAMI smartphone app through provided credentials. The app access is provided on the day of patient's hospital discharge; however, the app is not activated yet until the next day. When the nurses activate the specific app access (through the appcode in myFAMI web app), the participants get access to the intervention and can submit survey responses. The personalized study id for an individual participant is not shown in the myFAMI app.
- Survey notifications: Participants receive a daily reminder notification at 8:00 AM local time for the 30 days of intervention. The notification is shown by the myFAMI smartphone app and takes participants directly to the submit survey page. The notification is sent from Firebase Cloud Messaging (FCM) [56] servers through the myFAMI web server and is a key component of the myFAMI app framework. For every day, there is a personalized message for the notification (discussed in the Factors to Improve Efficiency section).
- Survey submission: The prime task of the participants in the myFAMI smartphone app is to complete the daily survey and submit it. The survey starts the next day after installation (day of patient's hospital discharge) for 30 consecutive days and contains 8 questions as part of the intervention. The questions have a clear progress bar on top to understand the progress level of the survey, a clear description, and colorful illustrations (collected from royalty-free image websites) relevant to the questions. Participants can submit once a day starting at 8:00 AM local time and cannot submit more than once a day. In this study, participants were encouraged to submit responses between 8:00 AM and 10:00 AM local time. After 30 days of the intervention, the app shut off submission of further surveys.

The daily survey for participants consists of 8 specific questions regarding the health status of pediatric transplant patients. The daily survey is based on myFAMI [34], which is built upon individual and family self‐management theory, with a focus on family self‐management of pediatric transplant recipients at home.

The first 5 questions focus on clinical symptoms (ie, fever, vomiting, diarrhea, pain, and illness) because these can result in hospital readmission. Family members have 3 response options (ie, yes, no, and don’t know). A response of yes or don’t know prompts a trigger alert for the nurse to call the family for further discussion and an additional question to gather more data and inform the trigger alert conversation with the family. The last 3 questions assess difficulty with coping, administering medications, and attending hospital appointments. These difficulty questions are measured with a Likert scale of 0 to 10, where 0 represents no difficulty and 10 represents a great deal of difficulty. Any response of 3 or greater prompts a trigger alert for the nurse to call the family for further discussion (trigger notification); see Table 2.

**Figure 9:**
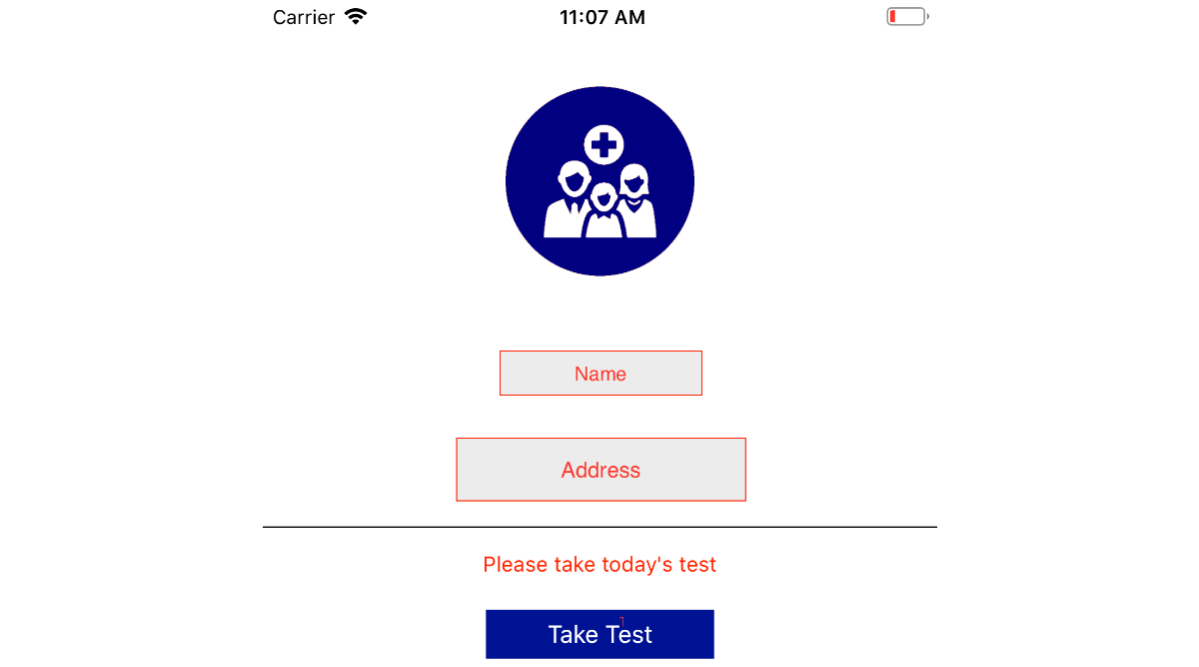
Homepage view of the app, with name and address redacted.

**Figure 10:**
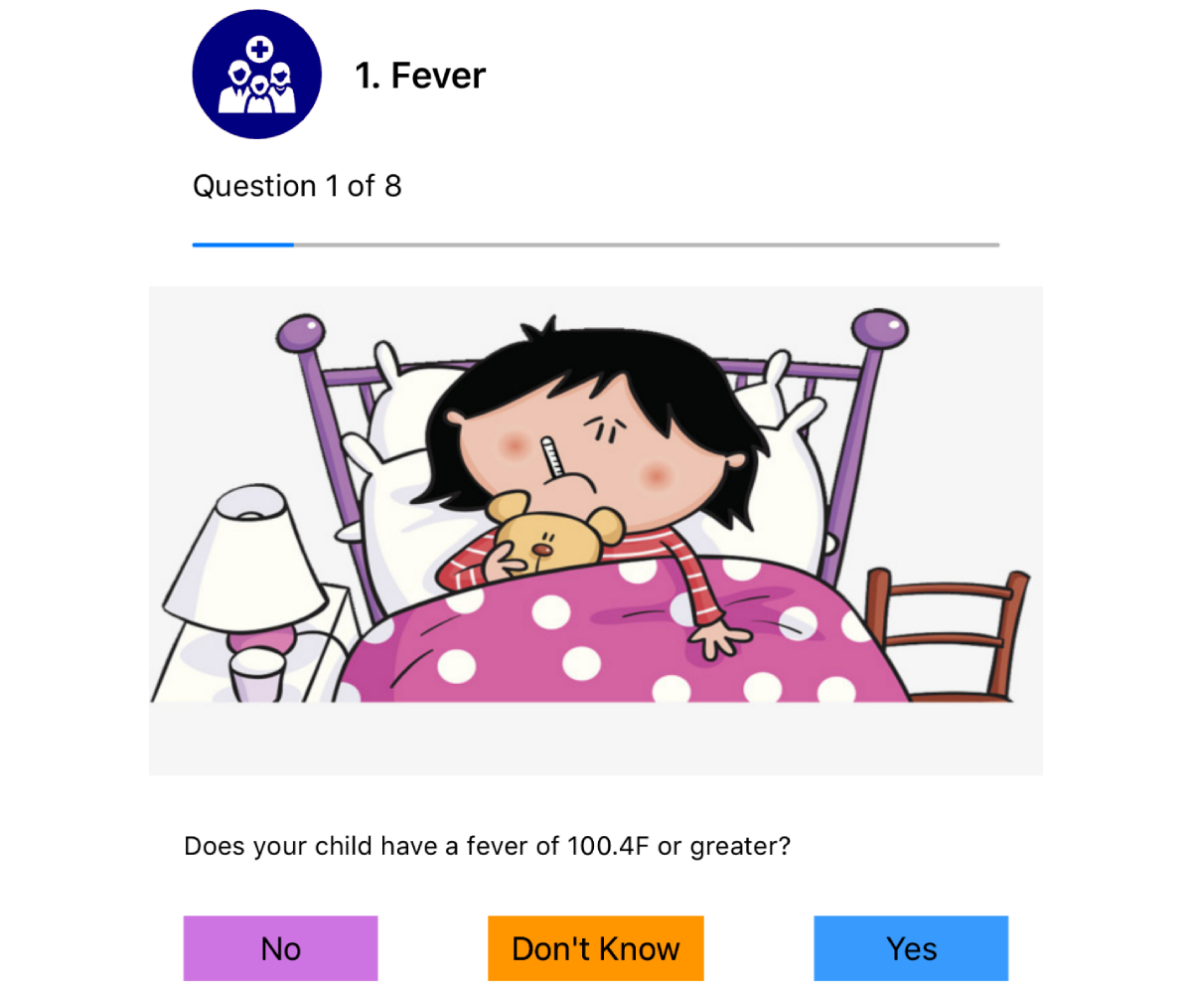
First question of the survey with specific question, relevant colorful illustration, and clear progress bar.

**Figure 11:**
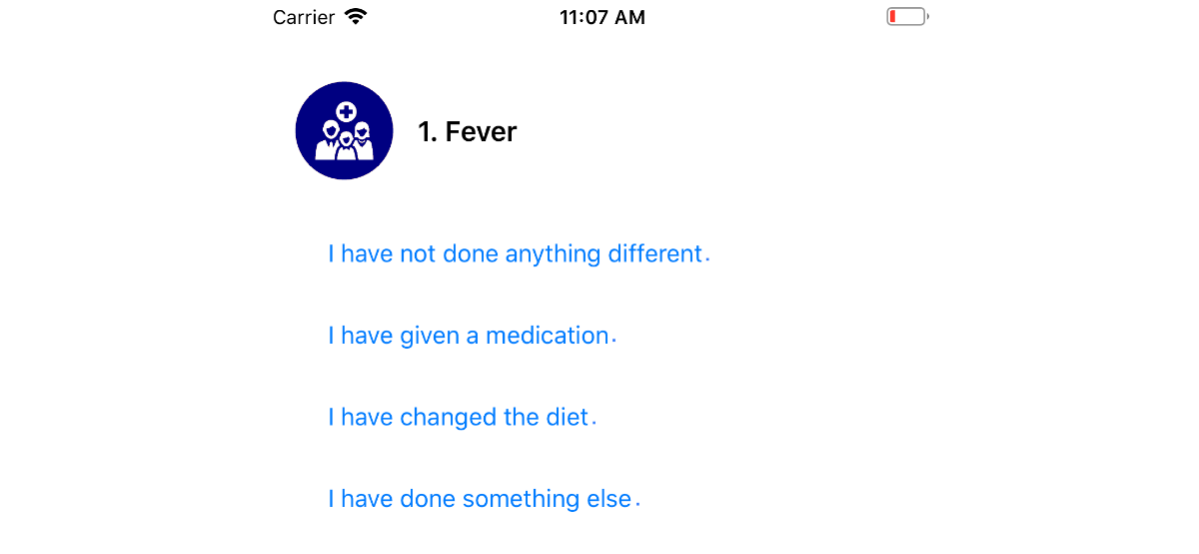
Interpretable responses as follow-up for selecting "Yes" or "Don’t know" in the previous question.

**Figure 12:**
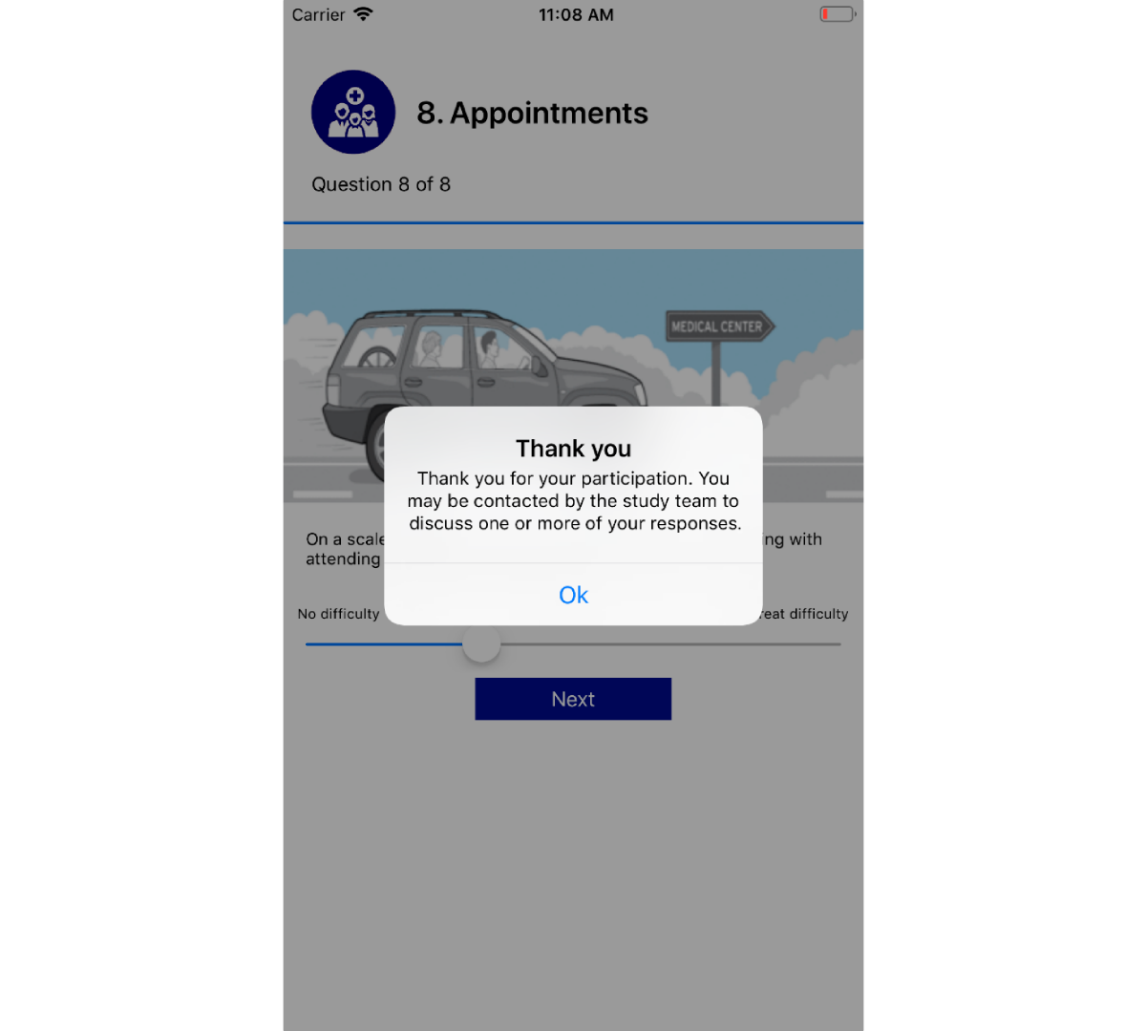
"Thank you" message after successful completion of survey submission.

An outline of individual questions, response options, and the rationale is discussed in detail [34]. Screenshots of selected pages of the myFAMI smartphone app are presented in Figures 9-12.

**Table 2:** Outline of individual questions in the myFAMIa smartphone app, with responses and trigger components.

Concern	Response type	Response options	Additional question (if answered yes or don’t know)	Trigger
Fever	3 survey options	Yes/no/don’t know	Please provide additional information (select only one option): (a) I have not done anything different. (b) I have administered a medication. (c) I have changed the diet. (d) I have done something else.	Yes/don’t know
Vomiting	3 survey options	Yes/no/don’t know	Please provide additional information (select only one option): (a) I have not done anything different. (b) I have administered a medication. (c) I have changed the diet. (d) I have done something else.	Yes/don’t know
Diarrhea	3 survey options	Yes/no/don’t know	Please provide additional information (select only one option): (a) I have not done anything different. (b) I have administered a medication. (c) I have changed the diet. (d) I have done something else.	Yes/don’t know
Pain	3 survey options	Yes/no/don’t know	Please provide additional information (select only one option): (a) I have not done anything different. (b) I have administered a medication. (c) I have changed the diet. (d) I have done something else.	Yes/don’t know
Illness	3 survey options	Yes/no/don’t know	Please provide additional information (select only one option): (a) I have not done anything different. (b) I have administered a medication. (c) I have changed the diet. (d) I have done something else.	Yes/don’t know
Coping	Likert scale	0 (no difficulty) to 10 (great deal of difficulty)	N/A^b^	≥3
Medication	Likert scale	0 (no difficulty) to 10 (great deal of difficulty)	N/A	≥3
Appointments	Likert scale	0 (no difficulty) to 10 (great deal of difficulty)	N/A	≥3

^a^myFAMI: mHealth family self-management intervention.

^b^N/A: not applicable.

A key component of the myFAMI app framework is the trigger alerts sent to the nurses. A preidentified response of yes or don’t know for the first 5 symptom-related questions or a response of 3 or greater to the last 3 coping-related questions in the myFAMI web server sends a trigger alert to the study nurses. A notification with participant details and the survey response is sent to the communication endpoints, including 2 emails and 2 pager numbers (PI and study nurses) for a prompt response. The trigger alert notifies the nurses and tracks the participants who may benefit from a phone call from one of the study nurses. A brief overview of the trigger alert process is also detailed in Figure 1.

## Discussion

### Principal Findings

We discussed the final design of the myFAMI app framework in detail, with a technical breakdown of the ecosystem. In this section, we present our principal findings from planning, designing, developing, and executing the myFAMI app framework. The general acceptability and usability portray the potential to involve more family members of pediatric transplant patients for further research. The major strength of our design process is the integration of the AR methodology with theory-based intervention [57], in the context of transplant patient care, along with the application of specific factors to improve efficiency in order to improve the usability of the app. However, there are minimal differences in the content and execution of the proposed framework, which were limitations of our integrated process.

### Strengths

The key strength of the research conducted for myFAMI is the process of integrating theory-based intervention [57] with the AR methodology [39,40]. myFAMI is the first of its kind intervention—the first RCT on the efficacy of a theory-based intervention on pediatric transplant patients’ and families’ postdischarge adjustment at home. The myFAMI app framework needed to be fluent and flawless to be able to handle the intervention over 4 major US-centered pediatric centers. We emphasized on adding action, reaction, and adjustment cycle to the software framework to fit in with the AR methodology, as well as prepare a robust app ecosystem for myFAMI.

Second, the identification and incorporation of factors to improve efficiency in the myFAMI app framework improved the overall user experience. Since the myFAMI app framework had 2 different sections (web app and smartphone app) and user groups (nurses and family members), we had to consider the unique user experiences, comfort, appropriate features, and communication format. Personal encouragement messages helped the participants remain on track with survey submissions as well as avoid the monotony of the daily survey submission. The focus was a friendly support system for the family. While completing the survey using the smartphone app, a clean and clear progress bar provided a sense of completion. Finally, the options for additional questions are framed in such a way that the survey feels like a conversation and the automation of the process seems less mechanical.

Another key strength of the design process was the iterative research discussions and opportunity to discuss concerns. The complete framework went through several software versions before the final MVP, which is in the essence of the AR methodology. For example, initially the image related to the question on diarrhea had an image with a person sitting on a toilet. Upon discussion, the image was replaced. The research meetings helped both the provider and computer scientists in planning a better software solution; the provider learned about the intricate process of software development and technology, and the computer scientists learned about a medical intervention conduction protocol. This reflexivity is one of the core components of the AR methodology and is generated in a better pool of knowledge, which can be impactful for future research as well.

Finally, automated visualization of aggregated survey responses was meaningful for the research team to monitor. A line chart for temporal data is a clean and straightforward visualization; however, it allowed for detecting a pattern out of individual pediatric patient responses. Our future exploration includes complex data visualization on aggregated patient subgroups.

### Limitations

Our proposed myFAMI app framework is highly effective in capturing responses for a smartphone-based intervention; however, there are limitations. As a software application, there were minor bugs identified during the deployment of the solution. Although alpha testing captured about 95% of commonly faced issues, new sets of issues arose when the app was deployed in the real world [58]. Even after rigorous testing, we faced random issues, including empty notifications triggered in the app. It is important to identify and resolve random bugs in the event notification prompting at the local time of the server, not the participant. One important limitation with app design was that pediatric transplant patient family members only provided feedback during beta testing. Transplant team members (nurses and doctors) were included in earlier development phases (ideation and alpha testing). Future projects should consider family member inclusions in the ideation phase of similar app development. Another key issue was to ensure that the survey response was not submitted accidentally. The protocol ensured the survey was actually submitted by the participant and not accidentally. To mitigate this, log-in credentials were required by the app for each submission; however, alpha testing showed that this reduced survey responses. Later, this security check was removed and a single prompt (“Are you sure you want to submit?”) was used to minimize accidental submissions.

### Future Directions

Our research completes a working prototype for myFAMI using smartphones; however, there are unexplored future directions that could improve the user experience as well as aid future researchers explore a similar problem domain. Primarily, for our myFAMI smartphone app, the survey questions would be randomized to reduce the risk of bias introduced by the survey question order [59] or survey fatigue [60]. Individual sets of images can be selected and shown in random in place of one single image for each question to avoid monotony. Additionally, different sets of colors or patterns of stripes for the myFAMI smartphone app need to be added as the color scheme, making it suitable for the color-blind population [61]. Second, for the myFAMI web app, better visualization tools (circular area chart or violin plot in place of a line plot) could be incorporated to improve the researchers’ experience. Finally, for myFAMI in general, a communication protocol for reconnection and encouragement can be established in case a participating family frequently misses survey submissions. Positive reinforcement is already added in the smartphone app (“You are doing great” after survey submission).

### Conclusion

In this paper, we presented an overview of the design and development of the myFAMI app framework. We discussed relevant factors to improve efficiency, development features, and general insights by conducting a smartphone-based intervention for families of pediatric transplant recipients. Based on the literature review, this is the first study that evaluates a smartphone-based intervention to improve family self-management for family members of children who received a heart, kidney, or liver transplant. Our study portrays a need for strong collaboration between computer scientists, expert providers, and nurses in addressing technical difficulties and making it a successful intervention through the AR methodology. This study also lays the foundation for researchers to carefully integrate necessary information (from relevant stakeholders, their experience, or the literature) to provide a robust and efficient solution and evaluate the acceptability, utility, and usability for similar studies in the future.

## Abbreviations

API: Application Programming Interface
mHealth: mobile health
MVP: minimum viable product
MVT: model-view-template
myFAMI: mHealth family self-management intervention
PI: principal investigator
RCT: randomized controlled trial
SDK: software development kit
